# Weight Perception, Weight Stigma Concerns, and Overeating

**DOI:** 10.1002/oby.22224

**Published:** 2018-06-28

**Authors:** Eugenia Romano, Ashleigh Haynes, Eric Robinson

**Affiliations:** ^1^ Institute of Psychology, Health & Society University of Liverpool Liverpool UK

## Abstract

**Objective:**

Perceiving one's own weight status as being overweight is a likely motivation for weight loss. However, self‐perceived overweight status has also been found to be associated with overeating and weight gain. This study examined whether weight stigma concerns explain why individuals who perceive their weight status as overweight are at increased risk of overeating.

**Methods:**

We conducted two survey studies of United States adults (*N* = 1,236) in which we assessed whether weight stigma concerns explain the cross‐sectional relationship between perceived overweight and overeating tendencies.

**Results:**

Across two studies, the cross‐sectional relationship between perceived overweight and overeating tendencies was in part explained by weight stigma concerns. Participants who perceived their weight as “overweight” reported greater weight stigma concerns than participants who perceived their weight as “about right,” and this explained 23.3% (Study 1) to 58.6% (Study 2) of the variance in the relationship between perceived overweight and overeating tendencies.

**Conclusions:**

Weight stigma concerns may explain why perceiving one's own weight status as overweight is associated with an increased tendency to overeat.

## Introduction

The failure of individuals with overweight to accurately identify their weight status has been highlighted as a cause for concern, as it is presumed that this failure might lead to ineffective weight management. In support of this idea, studies have demonstrated that self‐perception of overweight is associated with attempted weight loss and weight loss intentions among adults and adolescents of overweight status 1, 2, 3. However, recent findings have suggested that self‐perception of overweight is associated with worse weight management over time. Self‐perceived overweight is a risk factor for increased weight gain, both for adults and adolescents with normal weight and overweight status 4, 5, while weight status misperception among adolescents with overweight seems to be protective against weight gain 2. This may be partly explained by overeating. A study by Saules et al., for example, found self‐perceived overweight to be associated with binge eating among adults with normal weight and overweight 6, and a recent systematic review found evidence that self‐perception of overweight tends to be associated with disordered eating in participants with both normal weight and overweight or obesity 7.

One reason why self‐perception of overweight may be associated with overeating is because of the widespread stigma attached to larger body sizes 8, 9, which may lead to concerns over being negatively evaluated, rejected, or avoided because of body weight. In an experimental context, exposing participants to stigmatizing information about larger body sizes has been shown to promote increased food intake in women with overweight 10 and in women who perceived themselves as having overweight 11. In line with research on social anxiety and eating pathology in undergraduate students 12, 13, a potential explanation of these experimental findings is that awareness of weight stigma causes individuals who perceive themselves as having overweight to experience a fear of being stigmatized on the basis of their weight 14, regardless of whether they have previously experienced discrimination or mistreatment because of their body weight. These weight stigma concerns present a form of social identity threat, which has been shown to increase stress in women with overweight 15 and has been hypothesized to encourage overeating 16.

**Figure 1:**
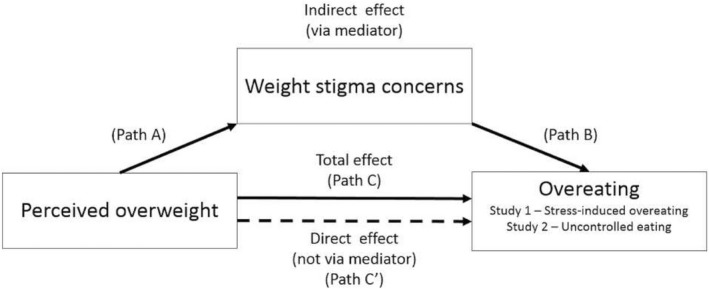
Hypothesized test of indirect effect.

Although self‐perception of overweight has now been shown to be associated with overeating among female adolescents 17 and young adult females with overweight 18, we are not aware of research that has attempted to explain the psychological mechanisms underlying this relationship. In the present research, we conducted two studies of United States adults to examine whether the cross‐sectional relationship between self‐perceived overweight and overeating tendencies is explained by heightened weight stigma concerns among individuals who perceive their weight status as overweight (Figure 1). We controlled for other factors that may confound the relationships of interest, including demographic and health variables and additional psychological variables (neuroticism and depression in Study 1 and 2; self‐esteem, body dissatisfaction, and physical activity in Study 2). This set of covariates was chosen because each has been demonstrated to be related to either perceived weight status 19, 20, 21, overeating 22, or both 23, 24, 25, 26, 27, 28. We additionally controlled for perceived weight discrimination in both studies because we were interested in isolating the effect of concerns over being stigmatized based on weight independently of the objective experience of weight‐based discrimination.

## Methods

### Study 1

#### Sample

Our analytic sample size in Study 1 provided sufficient power to detect small effect sizes (1 – β ≥ 0.80, α = 0.05, *f* ^2^ ≥ 0.02) for each pathway of our proposed test of indirect effect using bias‐corrected bootstrap models while accounting for covariates 29. A total of 718 United States adult participants were recruited via Amazon Mechanical Turk (https://www.mturk.com/) to complete an online questionnaire advertised as “weight and personal characteristics.” We decided a priori to exclude participants who failed at least one attention check (see online Supporting Information for full information on attention checks), who self‐perceived their weight as underweight (because few participants were likely to report this perception), or who reported weight and height data that produced an implausible BMI, using criteria as in previous research 30; 81 participants failed at least one attention check, 39 reported a self‐perception of underweight, and 11 reported implausible BMIs. This resulted in a final analytic sample of 587 participants. Sample characteristics are reported in Table 1.

**Table 1:** Sample characteristics

	Study 1	Study 2
**Age, mean (SD)**	36.47 (11.96)	38.73 (12.16)
**BMI, mean (SD)**	27.08 (6.03)	26.86 (5.59)
**Gender (% women)**	56.4	57.5
**Perceived overweight, %**	61.3	58.9
**Long‐standing illness (% yes)**	23.9	23.3
**Ethnicity, %**		
**White**	78.4	81
**Black**	7.2	7.1
**Asian**	5.8	4.9
**Hispanic**	5.1	4.2
**Mixed**	3.1	2.2
**Other**	0.5	0.6
**Annual income, %**		
**Less than $26,000**	31.7	28.4
**Between $26,000 and $39,999**	21.1	20.3
**Between $40,000 and $49,999**	14.5	13.6
**Between $50,000 and $74,999**	21.0	22.5
**Between $75,000 and $99,999**	7.3	8.9
**$100,000 or higher**	4.4	6.3
**Educational attainment, %**		
**Never completed high school**	0.3	0.3
**Completed high school**	43.1	36.7
**Bachelor's degree**	42.1	49.0
**Master's degree**	11.1	10.0
**PhD/professional degree**	3.4	4.0

#### Demographics

Participants reported their age, gender, ethnicity, current annual income, and highest education level. Participants also reported their height (feet and inches) and weight (pounds), which were converted to metric measures to calculate BMI (kilograms per meter squared). Presence of chronic illness was assessed with a single yes or no item.

#### Perceived weight

Consistent with previous research 4, participants were asked to describe their weight on a 6‐point scale (“very underweight,” “underweight,” “about the right weight,” “overweight,” “very overweight,” or “obese”). Based on their answers, participants were divided in the following two categories: perceived normal weight, for those who perceived themselves as “About the right weight” (representing the reference category), and perceived overweight, for those whose answers ranged from “Overweight” to “Obese.”

#### Weight stigma concerns

Weight stigma concerns were assessed using the Weight Stigma Concerns Scale 14. The scale consists of five items (e.g., “I am concerned that other people's opinion of me will be based on my weight”), to which participants indicate their agreement on 7‐point Likert scales ranging from 1 (“strongly disagree”) to 7 (“strongly agree”). Responses were summed, with higher values indicating greater weight stigma concerns. This scale had excellent internal consistency in the present study (α = 0.95).

#### Overeating tendencies

To assess overeating tendencies, participants completed a measure of stress‐induced overeating. Participants were asked to indicate the extent to which they typically engage in the following behaviors when stressed: “eating more than usual to enhance my mood” and “eating more of my favorite foods to enhance my mood” 4, 31. Participants responded to each item on 4‐point Likert scales ranging from 1 (“not at all”) to 4 (“a lot”), and the mean of the responses to the two items was calculated, with higher scores indicating a greater tendency toward stress‐induced overeating (α = 0.86 in present study).

#### Neuroticism

Participants completed the Neuroticism subscale of the Mini International Personality Item Pool 32. This brief measure has been psychometrically validated as a measure of the Big Five personality traits 32. The Neuroticism subscale had good internal consistency in the present study (α = 0.80).

#### Depressive symptoms

Depressive symptoms were assessed using the 10‐item Center for Epidemiological Studies Depression Scale 33. Internal consistency for the scale was good in the present study (α = 0.89).

#### Perceived weight discrimination

An adapted version of the Perceived Everyday Experiences with Discrimination Scale 14, 34 measured how frequently participants reported encountering a set of six discriminatory experiences in their daily lives because of their weight. In this study, the scale had good internal consistency (α = 0.89). See the online Supporting Information for additional details on included measures.

In Study 1 and Study 2, we collected additional self‐report questionnaires for the purpose of other research questions, and these are reported in full in the online Supporting Information.

#### Procedure

After providing informed consent, participants completed the demographics and perceived weight measures. Measures of weight stigma concerns, perceived weight discrimination, neuroticism, depression, and stress‐induced eating were then completed on randomized consecutive pages of the online survey. The survey included four attention checks, and participants who failed to answer them properly were screened out of the questionnaire. Participants were provided with a small monetary reward upon completion of the questionnaire. Ethical approval was obtained from the University of Liverpool Ethics Committee.

#### Data analysis

The PROCESS macro for SPSS (model 4; IBM, Corp., Armonk, New York) with 5,000 bootstrap samples 35 was used to test whether self‐perceived overweight (relative to perceived normal weight) had an indirect effect on stress‐induced overeating through weight stigma concerns. In our main analysis, all tests of indirect effects were adjusted for the following demographic and health variables: gender, age, BMI, ethnicity (white or not), income, education, and presence of chronic illness. As a test of robustness, we additionally controlled for neuroticism, perceived weight discrimination, and depression in a second analysis.

We also conducted two tests of conditional indirect effects using the PROCESS macro for SPSS (model 59) to test whether participant gender or the accuracy of perceived overweight (inaccurate perception of overweight [subsample of participants with BMI < 25], accurate perception of overweight [subsample of participants with BMI ≥ 25]) moderated the indirect effect of perceived overweight on stress‐induced overeating through weight stigma concerns in the fully adjusted analyses.

### Study 2

#### Sample

We powered Study 2 to be able to detect the effects observed in Study 1 (1 − β ≥ 0.80; α = 0.05; *f* ^2^ ≥ 0.03), oversampling for participant exclusions. We recruited 804 United States adults via Amazon Mechanical Turk to complete a study on “the relationship between weight, personal characteristics, and wellbeing.” Of the 804 participants, 97 were excluded because they failed an attention check, 40 perceived their weight as being underweight, 14 reported implausible BMIs, and 4 participants reported an age of <18 years old, leaving an analytic sample of 649.

#### Measures

Demographics, perceived weight, perceived weight discrimination, weight stigma concerns, neuroticism, and depressive symptoms were measured as in Study 1.

#### Overeating tendencies

The Uncontrolled Eating subscale of the three‐factor Eating Questionnaire‐Revised 18 36 was administered to assess the tendency to overeat. The subscale consists of nine items (e.g., “Sometimes when I start eating, I just can't seem to stop”) answered on 4‐point Likert scales, ranging from 1 (“definitely false”) to 4 (“definitely true”). The Uncontrolled Eating subscale has been validated against self‐reported food intake in a general population 37. Internal consistency was excellent in the present study (α = 0.91).

#### Physical activity

Physical activity was assessed using a single‐item measure (“In the past week, on how many days have you done a total of 30 minutes or more of physical activity, which was enough to raise your breathing rate? This may include sport, exercise, and brisk walking or cycling for recreation or to get to and from places, but should not include housework or physical activity that may be part of your job”), which has been validated against other widely used physical activity questionnaires, showing moderate positive correlations (*r* = 0.53) and a good test‐retest reliability (*r* = 0.72) 38.

#### Self‐esteem

Self‐esteem was assessed using Rosenberg's Self‐Esteem Scale 39. Internal consistency was excellent in the present study (α = 0.94).

#### Body dissatisfaction

Body dissatisfaction was assessed using the Body Dissatisfaction subscale of the Eating Disorder Inventory 40. The scale had excellent internal consistency in the present study (α = 0.91). See the online Supporting Information for additional details on included measures.

#### Procedure

The additional measures were presented in randomized order alongside the other randomized measures as in Study 1. Participants were provided with a small monetary reward upon completion of the questionnaire. Ethical approval was obtained from the University of Liverpool Ethics Committee.

#### Data analysis

Data analysis was identical to Study 1. However, in the second test of indirect effects, we controlled for neuroticism, perceived weight discrimination, depression, self‐esteem, body dissatisfaction, and physical activity.

## Results

### Study 1

Correlations between the variables are presented in Supporting Information Table S1. In our first model (Table 2, Model 1), weight perception was a significant predictor of weight stigma concerns (unstandardized coefficient, B = 3.28; SE = 0.55; *P* < 0.001), and in turn, weight stigma concerns were a significant predictor of stress‐induced overeating (B = 0.04; SE = 0.01; *P* < 0.001). Perceived overweight (relative to perceived normal weight) had a significant indirect effect on stress‐induced overeating via weight stigma concerns (bootstrap estimate = 0.13; SE = 0.03; 95% CI: 0.08 to 0.20), with weight stigma concerns explaining 31.8% of the variance in the relationship between perceived overweight and stress‐induced overeating. In the fully adjusted model (Table 2, Model 2), perceived overweight relative to perceived normal weight had a significant indirect effect on stress‐induced overeating via weight stigma concerns (bootstrap estimate = 0.08; SE = 0.02; 95% CI: 0.04 to 0.13), and weight stigma concerns explained 23.3% of the variance in the relationship between perceived overweight and stress‐induced overeating.

**Table 2:** Indirect effect of perceived overweight on stress‐induced overeating via weight stigma concerns (Study 1)

		Unstandardized coefficient	SE	*P*	Bootstrap 95% CI	Model *R* ^2^/proportion mediated (%)	Standardized coefficient^a^	SE	95% CI
**Model 1** b	**Path A**	3.28	0.55	<0.001	2.19 to 4.36	–	0.50	0.08	0.33 to 0.67
	**Path B**	0.04	0.01	<0.001	0.03 to 0.05	–	0.29	0.04	0.20 to 0.38
	**Indirect effect**	0.13	0.03	–	0.08 to 0.20	31.8%	0.14	0.03	0.09 to 0.22
	**Path C (total effect)**	0.41	0.08	<0.001	0.25 to 0.57	0.166	0.45	0.09	0.27 to 0.64
	**Path C' (direct effect)**	0.28	0.08	<0.001	0.12 to 0.44	0.223	0.31	0.09	0.12 to 0.49
**Model 2** c	**Path A**	2.66	0.51	<0.001	1.65 to 3.67	–	0.41	0.08	0.25 to 0.56
	**Path B**	0.03	0.01	<0.001	0.01 to 0.04	–	0.21	0.05	0.11 to 0.30
	**Indirect effect**	0.08	0.02	–	0.04 to 0.13	23.3%	0.08	0.03	0.04 to 0.14
	**Path C (total effect)**	0.33	0.08	<0.001	0.17 to 0.48	0.234	0.36	0.09	0.18 to 0.54
	**Path C' (direct effect)**	0.25	0.08	0.002	0.09 to 0.41	0.258	0.28	0.09	0.10 to 0.46

Indirect effect = effect of perceived overweight on stress‐induced overeating through weight stigma concerns; Path A = correlation between perceived overweight and weight stigma concerns; Path B = correlation between weight stigma concerns and stress‐induced overeating; Path C = effect of perceived overweight on stress‐induced overeating when weight stigma concerns are not present in the model; Path C' = correlation between perceived overweight and stress‐induced overeating after taking weight stigma concerns into account.

a Calculated by repeating analysis of indirect effects on *z* scores for all continuous variables (age, BMI, neuroticism, perceived weight discrimination, and depression).

b Adjusted for age, gender, ethnicity (white, nonwhite), income, education, chronic illness, and BMI.

c Adjusted for variables listed for Model 1 plus neuroticism, perceived weight discrimination, and depression.

For gender, the index of moderated mediation was not significant (bootstrap estimate = 0.07; SE = 0.04; CI: −0.01 to 0.15), suggesting that gender did not moderate the indirect effect of perceived overweight on stress‐induced overeating via weight stigma concerns. For weight perception accuracy, the index of moderated mediation was not significant (bootstrap estimate = −0.01; SE = 0.07; CI: −0.17 to 0.10), indicating that weight perception accuracy did not moderate the indirect effect of perceived overweight on stress‐induced overeating via weight stigma concerns.

Weight stigma concerns partially explained the relationship between perceived overweight status and overeating. A limitation of Study 1 was that although our measure of overeating has been shown to prospectively predict increased weight gain 4, it is a short form measure that has not been formally validated. We addressed this in Study 2 by using a validated measure of overeating that has been shown to be associated with increased energy intake 37, 41, the Uncontrolled Eating subscale of the Three‐Factor Eating Questionnaire‐Revised 18 36. Moreover, in Study 2, we controlled for further variables that we reasoned may be confounders of our proposed indirect pathway, namely, body dissatisfaction, self‐esteem, and physical activity.

### Study 2

Correlations between the variables are presented in Supporting Information Table S2. Results from our main analyses and relative standardized effects are presented in Table 3. In our first model (Table 3, Model 1), weight perception (perceived overweight relative to perceived normal weight) was a significant predictor of weight stigma concerns (B = 6.50; SE = 0.76; *P* < 0.001), and weight stigma concerns significantly predicted uncontrolled eating (B = 1.02; SE = 0.11; *P* < 0.001). There was a significant indirect effect of perceived weight on uncontrolled eating via weight stigma concerns (bootstrap estimate = 6.65; SE = 1.06; 95% CI: 4.81 to 8.99), and weight stigma concerns explained 58.6% of the variance in the relationship between perceived overweight and uncontrolled eating. In the fully adjusted model (Table 3, Model 2), perceived overweight, relative to perceived normal weight, had a significant indirect effect on uncontrolled eating via weight stigma concerns (bootstrap estimate = 1.44; SE = 0.52; 95% CI: 0.61 to 2.65), and weight stigma concerns explained 44.3% of the variance in the relationship between perceived overweight and uncontrolled eating.

**Table 3:** Indirect effect of perceived overweight on uncontrolled eating via weight stigma concerns (Study 2)

		Unstandardized coefficient	SE	*P*	Bootstrap 95% CI	Model *R* ^2^/proportion mediated (%)	Standardized coefficient^a^	SE	95% CI
**Model 1** b	**Path A**	6.50	0.76	<0.001	5.01 to 8.00	–	0.72	0.08	0.55 to 0.88
	**Path B**	1.02	0.11	<0.001	0.81 to 1.23	–	0.41	0.04	0.33 to 0.49
	**Indirect effect**	6.65	1.06	–	4.81 to 8.99	58.6%	0.29	0.05	0.21 to 0.40
	**Path C (total effect)**	11.36	2.18	<0.001	7.08 to 15.63	0.133	0.50	0.09	0.32 to 0.69
	**Path C' (direct effect)**	4.70	2.13	0.027	0.53 to 8.88	0.249	0.21	0.09	0.03 to 0.39
**Model 2** c	**Path A**	2.78	0.72	<0.001	1.37 to 4.19	–	0.31	0.07	0.16 to 0.45
	**Path B**	0.52	0.14	<0.001	0.25 to 0.79	–	0.21	0.05	0.11 to 0.31
	**Indirect effect**	1.44	0.52	–	0.61 to 2.65	44.3%	0.06	0.02	0.03 to 0.12
	**Path C (total effect)**	3.26	2.17	0.13	−1.01 to 7.52	0.294	0.14	0.10	−0.04 to 0.33
	**Path C' (direct effect)**	1.81	2.15	0.40	−2.43 to 6.06	0.312	0.08	0.10	−0.11 to 0.27

Indirect effect = effect of perceived overweight on uncontrolled eating through weight stigma concerns; Path A = correlation between perceived overweight and weight stigma concerns; Path B = correlation between weight stigma concerns and uncontrolled eating; Path C = effect of perceived overweight on uncontrolled eating when weight stigma concerns are not present in the model; Path C' = correlation between perceived overweight and uncontrolled eating after taking weight stigma concerns into account.

a Calculated by repeating analysis of indirect effects on *z* scores for all continuous variables (age, BMI, neuroticism, perceived weight discrimination, depression, self‐esteem, body dissatisfaction, and physical activity).

b Adjusted for variables listed for Study 1, Model 1.

c Adjusted for variables listed for Study 1, Model 2, plus self‐esteem, body dissatisfaction, and physical activity.

For gender, the index of moderated mediation was nonsignificant (bootstrap estimate = −0.89; SE = 0.82; CI: −2.68 to 0.57), suggesting that gender did not moderate the indirect effect of perceived overweight on uncontrolled eating via weight stigma concerns. For weight perception accuracy, the index of moderated mediation was not significant (bootstrap estimate = −0.12; SE = 1.21; CI: −3.24 to 1.79), indicating that weight perception accuracy did not moderate the indirect effect of perceived overweight on uncontrolled eating via weight stigma concerns.

## Discussion

Individuals who perceive their weight status as overweight are more likely to overeat and gain more weight than those who do not perceive their weight status as overweight 4, 7. We hypothesized that heightened weight stigma concerns because of the widespread stigma associated with larger body sizes 8, 9 explain why individuals who perceive their weight status as overweight are at an increased risk of overeating relative to those who perceive their weight as “about right.” Across two studies of United States adults, we found that weight stigma concerns partly explained the cross‐sectional relationship between self‐perceived overweight (relative to perceiving one's weight as about right) and self‐reported overeating tendencies. In both studies, weight stigma concerns explained a substantial proportion of the cross‐sectional association between weight perception and overeating in both our main analyses (32%‐59% of variance) and in analyses that accounted for a range of other related psychological variables, including previous experience of weight discrimination (23%‐44% of variance). This pattern of results was observed regardless of gender and whether self‐perception of overweight was accurate or inaccurate.

In line with Hunger et al. 14, our proposed explanation of these findings is that the awareness of the stigma attached to larger body sizes causes individuals who perceive their weight as overweight to experience greater concern over being negatively evaluated, rejected, or avoided by others because of their weight. Given the nature of the present research, we cannot infer why weight stigma concerns are associated with overeating tendencies, but there are plausible mechanisms. In Study 1, we measured stress‐induced overeating, and based on previous research, it is plausible that the stress associated with weight stigma concerns directly stimulates overeating 42. Alternatively, experiencing stress hampers self‐regulatory ability, which in turn results in unintended overeating 11. In Study 2, we measured uncontrolled eating, and there is evidence that episodes of uncontrolled eating may occur in response to negative emotions 43. Therefore, individuals who self‐identify as having overweight may overeat as a way of coping with their concerns of being stigmatized by others because of their perceived body size. A better understanding of why weight stigma concerns are associated with overeating tendencies among individuals who self‐identify as overweight would be valuable. Given that self‐perception of overweight has been shown to be associated with a range of negative health outcomes, including depressive symptoms 23 and suicidal ideation 44, an examination of whether weight stigma concerns also partly explain these other findings would be informative.

In both studies, weight stigma concerns only partially explained the cross‐sectional association between self‐perception of overweight and overeating. Therefore, it is likely that there are other factors explaining this relationship. Internalized weight stigma may be an important factor to consider in future research. Individuals who perceive their weight status as overweight may internalize negative stereotypes about larger body sizes, and this negative self‐perception may lead to emotional overeating 45. There are also plausible physiological mechanisms by which the stigma of obesity could result in increased energy intake among individuals who self‐perceive their weight status as overweight 46. We also did not find that our main results were moderated by participant gender or accuracy of weight perception. However, previous research has shown that women are more likely to expect social rejection because of their weight 15.

In the present research, we replicated our findings across two studies using different measures of overeating tendencies and found consistent results across analyses that controlled for a range of potentially confounding psychological variables. A limitation of the present studies is that they were cross‐sectional, which precludes causal inference. For example, we cannot rule out reverse causality in the relationships we tested, and it is possible that a third unmeasured variable may explain the observed pattern of findings. The measures of overeating tendencies used in Study 1 have been shown to prospectively predict weight gain 4, and the measure we used in Study 2 has been formally validated against an objective measurement of food intake 37. However, the measures were self‐reported, which may have introduced measurement bias. Moreover, differences between the two measures of overeating used may explain why weight stigma concerns explained a greater proportion of the association between weight perception and overeating in Study 2 than in Study 1. Replication of our findings using longitudinal or experimental designs that rely on objective measurements of eating behavior would be valuable.

## Conclusion

The results of these two cross‐sectional survey studies suggest that weight stigma concerns may explain why perceiving one's own weight status as overweight is associated with an increased tendency to overeat.
