# Supplementary material for: Weight Perception, Weight Stigma Concerns, and Overeating
Source: Obesity (Silver Spring). 2018 Jun 28;26(8):1365–71. doi: 10.1002/oby.22224 (PMC6221161; doi:10.1002/oby.22224)
Supplement: Supplementary file 1 [file OBY-26-1365-s001.docx]

**Weight perception, weight stigma concerns, and overeating**

**Online supplemental materials**

**Additional details**

**Study 1**

***Measures***

*Demographics*

Participants indicated their ethnicity (White, Black, Asian, Hispanic, Mixed, or Other), income (Less than $26,000, Between $26,000 and $39,999, Between $40,000 and $49,999, Between $50,000 and $74,999, Between $75,000 and $99,999, $100,000 or higher), and highest education level (Never completed High School, Completed High School, Bachelor Degree, Masters Degree, and PhD/Professional Degree). Presence of chronic illness was assessed with a single yes/no item *“Do you have any long-standing illness, disability or infirmity? By long-standing, we mean anything that has troubled you over a period of time, or that is likely to affect you over a period of time”.* When calculating BMI, values lower than 15 and higher than 50 were considered implausible, according to criteria used in previous research (1).

*Neuroticism*

The neuroticism subscale of the Mini International Personality Item Pool (Mini IPIP) (2) consists of 4 items (e.g., *“I get upset easily”*), to which participants indicated how accurately each statement reflects their personality on 5-point Likert scales ranging from 1 (*‘Very Inaccurate’*) to 5 (*‘Very Accurate’*). Responses to each item were summed to provide a neuroticism score, with higher values indicating higher neuroticism.

*Depressive symptoms*

Participants completed the 10-item Centre for Epidemiological Studies Depression Scale (CES-D) (3), with items (e.g., *“I was bothered by things that don’t usually bother me”*) rated on 4-point scales ranging from 1 (‘*Rarely’*) to 4 (*‘All of the time’*). Responses to each item were summed to provide a measure of depressive symptoms, and higher scores indicated higher levels of depressive symptoms.

*Perceived weight discrimination*

Participants completed an adapted version of the Perceived Everyday Experiences with Discrimination Scale (4,5) in which they indicated how frequently they had encountered six discriminatory experiences because of their weight (e.g., *“You are treated with less courtesy or respect than others”*) on 6-point Likert scales ranging from 1 (‘*Never’*) to 6 (‘*Almost every day’*). The total score was calculated by summing answers to each item.

*Attention checks*

The attention checks included in the questionnaire were *“You travelled back in time”*, *“When I'm upset, select response option sometimes for this question”*, *“Select somewhat agree as the response option for this question”* and *“Please disregard this as a question and select agree”*. Participants who did not select *‘Never’* for the first item and who did not select the indicated response options for the others were excluded from the analyses.

***Additional measures***

In Study 1, we collected additional self-report measures for the purpose of testing other research questions unrelated to the hypotheses of the present studies.

*Demographics:* Participants also reported their heaviest weight as an adult and their parents’ education level. Moreover, they were asked about how a doctor or a healthcare professional would perceive their weight, and whether they had ever been diagnosed as overweight by a doctor or a healthcare professional.

*People pleasing:* Participants completed a short self-devised scale to measure people pleasing tendencies. Participants indicated their agreement to 4 statements (e.g. *“I am the type of person that tries to avoid arguments and disagreements”*). Responses were indicated on a 7-point Likert scale ranging from 1 (‘*Strongly* *disagree’*) to 7 (*‘Strongly agree’*).

*Adverse childhood experiences*: Participants completed the Adverse Childhood Experiences questionnaire (6). Participants indicated whether or not they experienced a list of 10 experiences (e.g. *“Did a parent or other adult in the household often or very often push, grab, slap, or throw something at you? Or ever hit you so hard that you had marks or were injured?”*), with responses indicated as either “Yes” or “No”.

*Life satisfaction:* Participants completed the Satisfaction with Life Scale (7). The scale consists of five items (e.g. *“The conditions of my life are excellent”*) with responses indicated on a 7-point Likert scale ranging from 1 (‘*Strongly* *disagree’*) to 7 (*‘Strongly agree’*).

*Emotional dysregulation:* Participants completed the 36-item Difficulties in Emotional Regulation Scale (8). Items such as *“When I'm upset, I become angry with myself for feeling that way”* are answered on a 5-points Liker scale ranging from 1 (*‘Almost never (0-10%)’*) to 5 (*‘Almost always* *(91 – 100%)’*).

*Self-control*: Participants completed the brief Self-control Scale (9). The scale has 10 items (e.g. *“I get distracted easily”*), with answers ranging from 1 (*‘Not at all like me’*), to 5 (*‘Very much like me’*).

*Attachment styles*: Participants completed the revised 36-item Experiences in Close Relationships Scale (10). Items such as *“My romantic partner makes me doubt myself”* are rated on a 7-point Likert scale ranging from 1 (‘*Strongly* *disagree’*) to 7 (*‘Strongly agree’*).

Table S1.

Correlation matrix for the variables analysed in Study 1.

|  |  | 1 | 2 | 3 | 4 | 5 | | | 6 | 7 | | 8 | | | 9 | 10 | | 11 | | | 12 |  |
| --- | --- | --- | --- | --- | --- | --- | --- | --- | --- | --- | --- | --- | --- | --- | --- | --- | --- | --- | --- | --- | --- | --- |
|  | 1. Age | -- |  |  |  | |  |  | | |  | |  |  | | |  | |  |  | | |
|  | 2. Gender | -.011 |  |  |  | |  |  | | |  | |  |  | | |  | |  |  | | |
|  | 3. Ethnicity | .136^**^ | .022 |  |  | |  |  | | |  | |  |  | | |  | |  |  | | |
|  | 4. Income | .163^***^ | -.072 | .056 |  | |  |  | | |  | |  |  | | |  | |  |  | | |
|  | 5. Education | .061 | .024 | .014 | .317^***^ | |  |  | | |  | |  |  | | |  | |  |  | | |
|  | 6 Chronic illness | -.131^**^ | -.081 | -.022 | .146^***^ | | .077 |  | | |  | |  |  | | |  | |  |  | | |
|  | 7. BMI | .144^***^ | -.083^*^ | .080 | .004 | | -.092^*^ | -.228^***^ | | |  | |  |  | | |  | |  |  | | |
|  | 8. Weight perception | .147^***^ | .014 | .042 | -.013 | | -.044 | -.165^***^ | | | .562^***^ | |  |  | | |  | |  |  | | |
|  | 9. Weight stigma concerns | -.051 | .177^***^ | .082^*^ | -.072 | | -.104^*^ | -.195^***^ | | | .454^***^ | | .428^***^ |  | | |  | |  |  | | |
|  | 10. Stress-induced eating | -.092^*^ | .160^***^ | -.001 | -.108^**^ | | -.041 | -.177^***^ | | | .256^***^ | | .301^***^ | .414^***^ | | |  | |  |  | | |
|  | 11. Neuroticism | -.184^***^ | .088^*^ | .015 | -.167^***^ | | -.058 | -.211^***^ | | | .028 | | .106^*^ | .305^***^ | | | .308^**^ | |  |  | | |
|  | 12. Depression | -.151^***^ | -.019 | -.002 | -.221^***^ | | -.074 | -.295^***^ | | | .084^*^ | | .144^***^ | .295^***^ | | | .307^***^ | | .638^***^ |  | | |
|  | 13. Perceived weight discrimination | -.078 | .007 | -.038 | -.034 | | -.110^**^ | -.045 | | | .251^***^ | | .180^***^ | .443^***^ | | | .231^***^ | | .196^***^ | .293^***^ | | |

Categorical variables: Gender (1 = Male, 2 = Female), Ethnicity (0 = Not White, 1 = White), Chronic illness (1 = Yes, 2 = No), and Weight perception (1 = Normal weight, 2 = Overweight).

* *p* <.05 (2-tailed).

***p* <.01 (2-tailed).

****p* <.001 (2-tailed).

**Study 2**

***Measures***

*Self-esteem*

Rosenberg’s self-esteem scale (12) consists of 10 items (e.g. *“I feel that I am a person of worth, at least on an equal plane with others”*) to which participants rated their agreement on 4 point Likert-scales, ranging from 1 (*‘Strongly disagree’*), to 4 (*‘Strongly agree’*). Responses were summed to provide a self-esteem score, with higher scores indicating higher self-esteem.

*Body dissatisfaction*

The Body Dissatisfaction subscale of the Eating Disorder Inventory (EDI-BD) (13) consists of 9 items that assess feelings of discontentment with the shape and size of regions of the body that are typically of concern to people with eating disorders (e.g., stomach, hips, thighs, buttocks). Responses are provided on 6-point Likert scales ranging from 0 (*‘Never’*) to 5 (*‘Always’*). The total score is obtained by reverse-coding five items and summing them with the other ones, with higher scores indicating higher body dissatisfaction.

*Attention checks*

The attention checks included in the questionnaire were *“You are often bullied, please select Never for this question”*, *“I think my feet are alright, please select Often for this question”*, and *“My life is perfect as it is, please select Agree for this question”*. Participants who did not select the indicated response options for the items were excluded from the analyses.

***Additional Measures***

In Study 2 we collected one additional self-report measure, the Self-Objectification Questionnaire (14), for the purpose of other research questions. Participants were asked to rank a list of ten body attributes (e.g. *“Strength”*, *“Physical Attractiveness”*) from the one which had the greatest impact on their physical self-concept (ranking it as *‘1’*), to the one which had the least impact on their physical self-concept (ranking it as *‘10’*).

Table S2.

Correlation matrix for the variables analysed in Study 2.

|  | 1 | 2 | 3 | 4 | 5 | 6 | 7 | 8 | 9 | 10 | 11 | 12 | 13 | 14 | 15 |
| --- | --- | --- | --- | --- | --- | --- | --- | --- | --- | --- | --- | --- | --- | --- | --- |
| 1. Age | -- |  |  |  |  |  |  |  |  |  |  |  |  |  |  |
| 2. Gender | .041 |  |  |  |  |  |  |  |  |  |  |  |  |  |  |
| 3. Ethnicity | .148^***^ | .085^*^ |  |  |  |  |  |  |  |  |  |  |  |  |  |
| 4. Income | -.015 | -.085^*^ | -.007 |  |  |  |  |  |  |  |  |  |  |  |  |
| 5. Education | .032 | -.097^*^ | -.003 | .329^***^ |  |  |  |  |  |  |  |  |  |  |  |
| 6. Chronic Illness | -.191^***^ | -.134^**^ | -.089^*^ | .212^***^ | .136^**^ |  |  |  |  |  |  |  |  |  |  |
| 7. BMI | .079^*^ | -.107^**^ | .054 | -.064 | -.141^***^ | -.153^***^ |  |  |  |  |  |  |  |  |  |
| 8. Weight perception | .087^*^ | .060 | .003 | -.009 | -.014 | -.171^***^ | .575^***^ |  |  |  |  |  |  |  |  |
| 9. Weight stigma concerns | -.107^**^ | .134^**^ | .054 | -.031 | -.059 | -.120^**^ | .406^***^ | .481^***^ |  |  |  |  |  |  |  |
| 10. Uncontrolled eating | -.145^***^ | .022 | .049 | -.018 | .034 | -.046 | .229^***^ | .295^***^ | .472^***^ |  |  |  |  |  |  |
| 11. Neuroticism | -.124^**^ | .175^***^ | .004 | -.122^**^ | -.059 | -.102^**^ | .106^**^ | .165^***^ | .371^***^ | .340^***^ |  |  |  |  |  |
| 12. Depression | -.083^*^ | .079^*^ | .022 | -.181^***^ | -.038 | -.228^***^ | .169^***^ | .243^***^ | .514^***^ | .398^***^ | .687^***^ |  |  |  |  |
| 13. Perceived weight discrimination | -.125^**^ | .002 | -.068 | .007 | -.057 | -.100^*^ | .211^***^ | .197^***^ | .516^***^ | .325^***^ | .249^***^ | .419^***^ |  |  |  |
| 14. Physical activity | .008 | -.149^***^ | -.004 | .122^**^ | .109^**^ | .114^**^ | -.200^***^ | -.199^***^ | -.220^***^ | -.171^***^ | -.259^***^ | -.268^***^ | -.040 |  |  |
| 15. Self-esteem | .113^**^ | -.034 | -.011 | .181^***^ | .089^*^ | .120^**^ | -.162^***^ | -.197^***^ | -.490^***^ | -.338^***^ | -.612^***^ | -.713^***^ | -.359^***^ | .296^***^ |  |
| 16. Body dissatisfaction | .030 | .278^***^ | .087^*^ | -.049 | -.075 | -.193^***^ | .453^***^ | .591^***^ | .623^***^ | .422^***^ | .363^***^ | .428^***^ | .304^***^ | -.315^***^ | -.461^***^ |

Categorical variables: Gender (1 = Male, 2 = Female), Ethnicity (0 = Not White, 1 = White), Chronic illness (1 = Yes, 2 = No), and Weight perception (1 = Normal weight, 2 = Overweight).

* *p* <.05 (2-tailed).

***p* <.01 (2-tailed).

****p* <.001 (2-tailed).

**References**

1. Armour C, Mullerova J, Fletcher S, Lagdon S, Burns CR, Robinson M, et al. Assessing childhood maltreatment and mental health correlates of disordered eating profiles in a nationally representative sample of English females. S*oc Psychiatry Psychiatr Epidemiol* 2016; 51(3): 383–93.

2. Donnellan MB, Oswald FL, Baird BM, Lucas RE. The mini-IPIP scales: tiny-yet-effective measures of the Big Five factors of personality. *Psychol Assess* 2006; 18(2): 192.

3. Radloff LS. The CES-D Scale: A Self Report Depression Scale for Research in the General Population. *Appl Psych Meas* 1977; 1(3): 385–401.

4. Hunger JM, Major B. Weight stigma mediates the association between BMI and self-reported health. *Health Psychol* 2015; 34(2): 172–5.

5. Williams DR, Yu Y, Jackson JS, Anderson NB. Racial differences in physical and mental health: Socio-economic status, stress and discrimination. *J Health Psychol* 1997; 2(3): 335–51.

6. Felitti VJ, Anda RF, Nordenberg D, Williamson DF, Spitz AM, Edwards V, et al. Relationship of childhood abuse and household dysfunction to many of the leading causes of death in adults: The adverse childhood experiences (ACE) study. A*m J Prev Med* 1998; 14(4): 245–58.

7. Diener E, Emmons RA, Larsen RJ, Griffin S. The Satisfaction With Life Scale. *J Pers Assess* 1985; 49(1): 71–5.

8. Gratz KL, Roemer L. Multidimensional Assessment of Emotion Regulation and Dysregulation: Development, Factor Structure, and Initial Validation of the Difficulties in Emotion Regulation Scale. *J Psychopathol Behav Assess* 2004; 26(1): 41–54.

9. Tangney JP, Baumeister RF, Boone AL. High Self-Control Predicts Good Adjustment, Less Pathology, Better Grades, and Interpersonal Success. *J Pers* 2004; 72(2): 271–324.

10. Fraley RC, Waller NG, Brennan KA. An item response theory analysis of self-report measures of adult attachment. *J Pers Soc Psychol* 2000; 78(2): 350–65.

11. Milton K, Bull FC, Bauman A. Reliability and validity testing of a single-item physical activity measure. *Br J Sports Med* 2011; 45(3): 203–8.

12. Rosenberg M. *Society and the Adolescent Self-Image*. Princeton, NJ, Princeton University Press; 1965.

13. Garner DM, Olmstead MP, Polivy J. Development and validation of a multidimensional eating disorder inventory for anorexia nervosa and bulimia. I*nt J Eat Disord* 1983; 2(2): 15–34.

14. Noll SM, Fredrickson BL. A Mediational Model Linking Self-Objectification, Body Shame, and Disordered Eating. *Psychol Women Quart* 1998; 22(4): 623–36.
